# Procalcitonin is not sufficiently reliable to be the sole marker of neonatal sepsis of nosocomial origin

**DOI:** 10.1186/1471-2431-6-16

**Published:** 2006-05-18

**Authors:** José B López Sastre, David Pérez Solís, Vicente Roqués Serradilla, Belén Fernández Colomer, Gil D Coto Cotallo, Xavier Krauel Vidal, Eduardo Narbona López, Manuel García del Río, Manuel Sánchez Luna, Antonio Belaustegui Cueto, Manuel Moro Serrano, Alfonso Urbón Artero, Emilio Álvaro Iglesias, Ángel Cotero Lavín, Eduardo Martínez Vilalta, Bartolomé Jiménez Cobos, "Grupo de Hospitales Castrillo"

**Affiliations:** 1 Service of Neonatology, Hospital Universitario Central de Asturias, Oviedo, Spain; 2 Service of Neonatology, Hospital Universitario La Fe, Valencia, Spain; 3 Service of Neonatology, Hospital Sant Joan de Déu, Barcelona, Spain; 4 Service of Neonatology, Hospital Universitario San Cecilio, Granada, Spain; 5 Service of Neonatology, Hospital Regional Universitario Carlos Haya, Málaga, Spain; 6 Service of Neonatology, Hospital Universitario Gregorio Marañón, Madrid, Spain; 7 Service of Neonatology, Hospital Universitario Doce de Octubre, Madrid, Spain; 8 Service of Neonatology, Hospital Clínico San Carlos, Madrid, Spain; 9 Service of Pediatrics, Complejo Hospitalario de la Seguridad Social, Segovia, Spain; 10 Service of Pediatrics, Hospital de León, León, Spain; 11 Service of Neonatology, Hospital de Cruces, Barakaldo, Spain; 12 Service of Neonatology, Hospital Universitario Virgen de la Arrixaca, Murcia, Spain; 13 Service of Neonatology, Hospital General Universitario de Alicante, Alicante, Spain

## Abstract

**Background:**

It has recently been suggested that serum procalcitonin (PCT) is of value in the diagnosis of neonatal sepsis, with varying results. The aim of this prospective multicenter study was to assess the usefulness of PCT as a marker of neonatal sepsis of nosocomial origin.

**Methods:**

One hundred infants aged between 4 and 28 days of life admitted to the Neonatology Services of 13 acute-care teaching hospitals in Spain over 1-year with clinical suspicion of neonatal sepsis of nosocomial origin were included in the study. Serum PCT concentrations were determined by a specific immunoluminometric assay. The reliability of PCT for the diagnosis of nosocomial neonatal sepsis at the time of suspicion of infection and at 12–24 h and 36–48 h after the onset of symptoms was calculated by receiver-operating characteristics (ROC) curves. The Youden's index (sensitivity + specificity - 1) was used for determination of optimal cutoff values of the diagnostic tests in the different postnatal periods. Sensitivity, specificity, and the likelihood ratio of a positive and negative result with the 95% confidence interval (CI) were calculated.

**Results:**

The diagnosis of nosocomial sepsis was confirmed in 61 neonates. Serum PCT concentrations were significantly higher at initial suspicion and at 12–24 h and 36–48 h after the onset of symptoms in neonates with confirmed sepsis than in neonates with clinically suspected but not confirmed sepsis. Optimal PCT thresholds according to ROC curves were 0.59 ng/mL at the time of suspicion of sepsis (sensitivity 81.4%, specificity 80.6%); 1.34 ng/mL within 12–24 h of birth (sensitivity 73.7%, specificity 80.6%), and 0.69 ng/mL within 36–48 h of birth (sensitivity 86.5%, specificity 72.7%).

**Conclusion:**

Serum PCT concentrations showed a moderate diagnostic reliability for the detection of nosocomial neonatal sepsis from the time of suspicion of infection. PCT is not sufficiently reliable to be the sole marker of sepsis, but would be useful as part of a full sepsis evaluation.

## Background

Infections of nosocomial origin are one of the most serious problems in modern neonatal units. Nursery-acquired infections are associated with increased mortality rates, prolonged duration of hospitalization in survivors, and high patient care expenditures. Because very low birth weight (VLBW) infants in neonatal intensive care units are at high risk for nursery-acquired infections, the frequency of these infections has increased in recent decades as a result of increased survival of immature neonates [1]. In a previous study of 30,993 admissions to neonatal units of 27 acute-care hospitals in Spain, the nosocomial sepsis rate was 2.1% with an incidence density of 0.89 per 1000 patient days. Sepsis rate was 15.6% among VLBW infants and 1.16% among those weighing ≥ 1500 g [2].

The prevention and control of these infections are thus major challenges for neonatal intensive care units (NICUs). Rapid diagnosis, however, is problematic because the earliest signs of nosocomial infection may be minimal and are similar to those of various noninfectious conditions. Bacterial cultures are time-consuming and other laboratory tests are either not available for routine use or lack sensitivity or specificity. In this situation, neonates with risk factors for infection or clinical suspicion of infection are empirically treated with antibiotics. On the other hand, the presence of multiresistant nursery flora complicates the choice of antimicrobials. To avoid unnecessary treatment of noninfected neonates, an early, sensitive and specific laboratory test would be helpful to guide clinicians in neonatal units in deciding whether or not to start antibiotics. Over the past few decades, several markers of neonatal infection especially leukocyte indexes and acute-phase reactants, some of which are commonly used in clinical practice, have been studied. However, there is less information on the value of these markers for the diagnosis of sepsis of nosocomial origin compared with neonatal sepsis of vertical transmission, and, to date, no single laboratory test has provided rapid and reliable identification of early infected neonates. This inability has led to search for new diagnostic markers [3,4].

It has been recently reported that procalcitonin (PCT), the prohormone of calcitonin, increases markedly in septic conditions [5] and has appeared to be a good predictor of infection severity. Furthermore, the finding that PCT is released into the circulation within 3 h after endotoxin injection, plateaus at 6 h, and remains elevated for 24 h, makes PCT a promising new agent for early and sensitive identification of severe infection both in adults and children with promising results [6]. The results of recent studies suggested the usefulness of PCT for early diagnosis of early-onset [7-19] and late-onset neonatal sepsis [19-23].

This objective of this prospective multicenter study was to assess the diagnostic usefulness of PCT as a single marker of neonatal sepsis of nosocomial origin.

## Methods

Since 1995 the neonatal services of 28 acute-care teaching hospitals distributed across 10 Autonomous Communities in Spain ("Grupo de Hospitales Castrillo") have been involved in an ongoing prospective surveillance project to assess the incidence and characteristics of nosocomial infections in the neonatal period [2,24]. The neonatal services of 13 hospitals participated in the present study. Between January 2000 and January 2001 all consecutive neonates aged between 4 and 28 days of life with clinical suspicion of sepsis of nosocomial origin were prospectively included in the study if blood samples were available for timed PCT measurement according to three postnatal periods: at the time of appearance of the first clinical manifestations and within 12–24 h and 36–48 h after the onset of symptoms, and complete neonatal and outcome data were collected to classify the infants into two distinct populations: confirmed sepsis and not confirmed sepsis. The study was approved by the Ethics Committees of the participating hospitals and the parents gave their informed consent.

Sepsis of nosocomial origin was suspected in the presence of at least three clinical signs and one risk factor for the nosocomial origin of the infectious process (as shown in Table 1), and laboratory signs consistent with infection (abnormal hematologic values and/or C-reactive protein > 1.2 mg/dL). Diagnostic of confirmed nosocomial sepsis was established when blood culture was positive. If the pathogens isolated in blood culture were traditional pathogens of vertical transmission (*Streptococcus agalactiae*, *Escherichia coli*) and there was a positive maternal vaginal culture with the same pathogen, the episode was considered of vertical transmission and was excluded from the study.

**Table 1:** Clinical signs of neonatal sepsis and risk factors for nosocomial transmission

Clinical signs	Data
Respiratory	Tachypnea (> 70 breaths/min in preterm babies, > 60 breaths/min in neonates at term)
	Subcostal and/or intercostal retractions
	Grunting
	Apnea > 10 seconds
	
Hemodynamic	Cyanosis
	Pallor
	Hypotension (blood pressure < 2 SD of the mean for age and weight)
	Tachycardia (> 180 beats/min)
	Bradycardia (< 100 beats/min in preterm babies, < 80 beats/min in neonates at term)
	Poor peripheral perfusion

Digestive	Rejection of food
	Vomiting
	Abdominal distention
	Hepatomegaly

Hematologic	Anemia (hemoglobin < 10 g/dL in preterm babies, < 11 g/dL in neonates at term)
	Jaundice (yellowish staining)
	Petechiae
	Echymoses

Risk factors for infection	Endotracheal intubation
	Central venous catheter
	Parenteral nutrition
	Nasogastric tube
	Urinary catheter
	Use of methylxantines
	Postnatal use of corticosteroids
	Ventriculoperitoneal shunt catheter
	Artificial feeding
	Previous surgical operation

Two successive positive blood cultures from peripheral percutaneous specimens were required for the diagnosis of coagulase-negative staphylococci (CoNS) infection [2]. Only one peripheral blood culture was considered valid when both bottles of the blood culture set grew isolates of CoNS organisms with identical antibiotic susceptibility patterns. In patients with a central line, criteria for CoNS infection included recovery of the same CoNS with identical antibiotic susceptibility patterns from the peripheral blood culture and culture of the catheter tip using the semi-quantitative method of Maki et al. [25]. Because quantitative cultures without removal of the catheter was not a routine microbiologic method in all participating hospitals, the increased bacterial colony counts from blood drawn through the suspected infected device compared to blood from a peripheral venipuncture was not required for diagnosis. For all blood cultures it was recommended to submit an amount of ≥ 1 mL to the laboratory.

### PCT assay

Blood samples were centrifuged within 30 min of collection. Serum was stored at -20°C before analysis. PCT was measured in duplicate by a specific immunoluminometric assay (LUMItest^®^, Brahms Diagnostica GmbH, Berlin, Germany), requiring 20 μL of serum and 2 h to complete. The limit of detection of this immunoluminometric assay is 0.08 ng/mL. Luminescence was measured automatically on a Lumat LB 9507 tube luminometer (Berthold Technologies GmbH & Co. KG, Bad Wildbad, Germany). All PCT assays were performed by two centralized clinical chemistry laboratories of two participating hospitals.

### Statistical analysis

Statistical analysis was done with the Statistical Package for the Social Sciences (SPSS) version 11.0 (SPSS Inc., Chicago, Ill, USA) and EPIDAT version 3.0 (Epidemiological Analysis of Tabulated Data developed by Servicio de Información sobre Saúde Pública de la Consellería de Sanidade de la Xunta de Galicia, Spain, and theHealth Situation Analysis Program of the Pan American Health Organization, Washington D.C., USA). PCT values are expressed as median and interquartile (25th–75th) range. Neither PCT values nor their log transformation were normally distributed, thus non-parametric tests were used for analysis. Comparison between groups was made with the Mann-Whitney *U *test. The reliability of serum PCT concentration for the diagnosis of neonatal sepsis of nosocomial origin was calculated by receiver-operating characteristics (ROC) curves. The Youden's index (sensitivity + specificity - 1) was used for determination of optimal cutoff values of the diagnostic tests in the different postnatal periods. Sensitivity, specificity, and the likelihood ratio of a positive and negative result with the 95% confidence interval (CI) were calculated. Statistical significance was set at *P *< 0.05.

## Results

A total of 100 neonates (57 males, 43 females) with clinically suspected nosocomial sepsis and a median (interquartile range) age of 13.6 (10.0–24.8) days were included in the study. The median (interquartile range) weight at birth was 1270 (950–1990) g and the gestational age 29.5 (27–34) weeks. Nosocomial neonatal sepsis was confirmed in 61 infants. Causative pathogens were as follows: coagulase-negative *Staphylococcus *in 22 patients, *Klebsiella pneumoniae *in 14, *Escherichia coli *in 7, *Enterobacter cloacae *in 6, *Enterococcus *in 5, *Candida *spp. in 2, *Pseudomonas aeruginosa *in 2, *Klebsiella oxytoca *in 1, *Staphylococcus aureus *in 1, and mixed infection by *K. pneumoniae *and *Enterococcus *in 1. Not confirmed nosocomial sepsis group included 7 patients with only one blood culture positive to CoNS that did not me meet the rest of our criteria for confirmed nosocomial sepsis.

**Figure 1:**
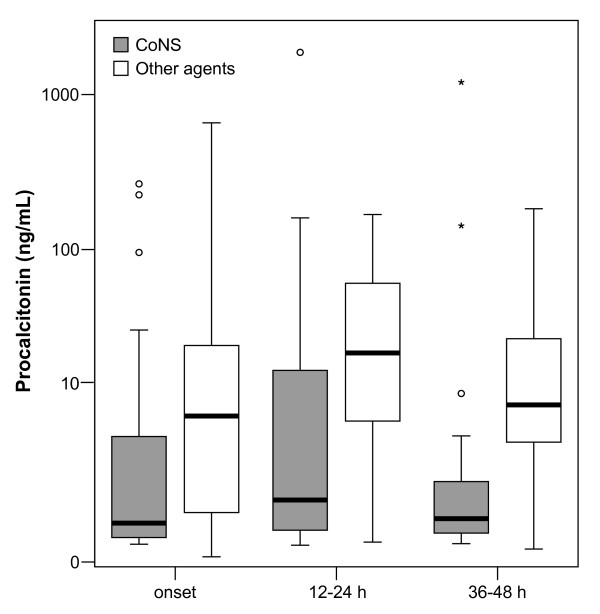
**PCT in sepsis caused by CoNS versus other pathogens**. Box plot comparing PCT concentrations in neonates with nosocomial sepsis caused by coagulase-negative *Staphylococcus *versus other causative pathogens.

As shown in Table 2, serum concentrations of PCT were significantly higher at initial suspicion and at 12–24 h and 36–48 h after the onset of symptoms in neonates with culture-proven sepsis than in neonates with clinically suspected but not confirmed sepsis. In the group of 61 infants with confirmed nosocomial sepsis, serum PCT concentrations were significantly lower in the group of 22 patients with infections caused by CoNS than in the remaining 39 patients with infections of other etiologies (Table 2, Figure 1).

**Table 2:** Differences in serum PCT values according to final diagnosis (confirmed vs. not confirmed) and etiology (coagulase-negative staphylococci vs. other pathogens) of sepsis

Study group	Serum PCT values, ng/mL median (25th–75th interquartile range)		
	
	Onset of symptoms	12–24 h	36–48 h
Not confirmed sepsis, n = 39	0.34 (0.24–0.58)	0.44 (0.25–1.20)	0.47 (0.33–0.92)
Confirmed sepsis, n = 61	2.46 (0.68–23.20)	12.65 (1.01–49.00)	5.15 (0.99–16.23)
*P *value*	< 0.0001	< 0.0001	< 0.0001
Sepsis caused by CoNS, n = 22	0.76 (0.42–7.70)	1.48 (0.56–16.44)	0.88 (0.51–2.27)
Sepsis caused by other pathogens, n = 39	7.60 (1.01–27.01)	20.85 (6.54–63.50)	8.62 (4.32–32.0)
*P *value*	< 0.049	< 0.003	< 0.0003

*Mann-Whitney *U *test.

**Figure 2:**
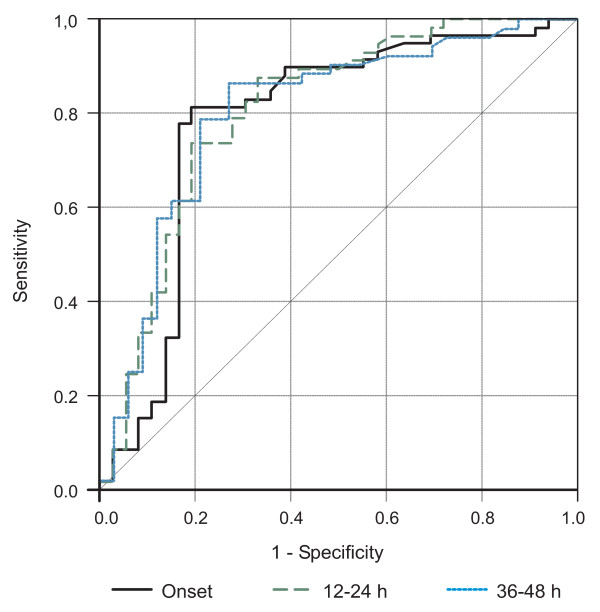
**ROC curves**. ROC curves of PCT at the time of clinical suspicion of nosocomial sepsis and at 12–24 h and 36–48 h after the onset of symptoms.

ROC curves comparing PCT values for diagnosis of neonatal sepsis of nosocomial origin at the time of clinical suspicion and 12–24 h and 36–48 h after the onset of symptoms are depicted in Figure 2. The areas under the ROC curve for the three periods were 0.783 (95% CI, 0.675 to 0.892), 0.805 (95% CI, 0.707 to 0.903), and 0.802 (95% CI, 0.699 to 0.905), respectively. Statistically significant differences in the areas under the ROC curves were not found. Cutoff levels with the optimum diagnostic efficiency derived from the ROC curves were ≥ 0.59 ng/mL at the time of clinical suspicion (sensitivity 81.4%, specificity 80.6%); ≥ 1.34 ng/mL at 12–24 h after the onset of symptoms (sensitivity 73.7%, specificity 80.6%); and ≥ 0.69 ng/mL at 36–48 h after the onset of symptoms (sensitivity 86.5%, specificity 72.7%) (Table 3).

**Table 3:** Diagnostic accuracy of serum PCT for the diagnosis of neonatal sepsis of nosocomial origin

Data	Onset of symptoms	12–24 h	36–48 h
Cutoff value, ng/mL	0.59	1.34	0.69
Sensitivity, % (95% CI)	81.4 (69.6–89.3)	73.7 (61.0–83.4)	86.5 (74.7–93.3)
Specificity, % (95% CI)	80.6 (65.0–90.2)	80.6 (65.0–90.2)	72.7 (55.8–84.9)
Positive predictive value, % (95% CI)	87.3 (76.0–93.7)	85.7 (73.3–92.9)	83.3 (71.3–91.0)
Negative predictive value, % (95% CI)	72.5 (57.2–83.9)	65.9 (51.1–78.1)	77.4 (60.2–88.6)
Youden's index	0.62	0.54	0.59
Likelihood ratio of a positive result (95% CI)	4.18 (2.13–8.23)	3.79 (1.91–7.50)	3.17 (1.80–5.60)
Likelihood ratio of a negative result (95% CI)	0.23 (0.13–0.40)	0.33 (0.21–0.52)	0.19 (0.09–0.38)

## Discussion

Sepsis of vertical transmission in the newborn infant has been a nearly constant concern in Neonatology, but development of nosocomial infections from acquisition of bacterial and other microbial pathogens in the nursery has become a challenging complication as a result of increased survival of VLBW infants or premature infants affected with severe diseases. However, there are relatively few studies of the value of serum PCT for the diagnosis of nosocomial neonatal sepsis, and none of them is a multicenter one. Monneret et al. [19] assessed daily variations in serum PCT in comparison with C-reactive protein (CRP) in 94 control and infected newborn infants, with 14 infected infants and 17 controls between the 4 and 28 days of life. Although in this study, procalcitonin seems to be an interesting marker of neonatal sepsis (early PCT peak compared with CRP), data to estimate diagnostic reliability are lacking. Chiesa and associates [9,21] reported the results of a study that included 23 cases of nosocomial infection and 92 asymptomatic controls between 3 and 30 days of life, in which serum PCT concentration discriminated all cases of sepsis with a 100% sensitivity and specificity. All infected infants had PCT values of ≥ 2 ng/mL and all controls ≤ 1 ng/mL. Enguix et al. [20] evaluated serum PCT as a diagnostic marker of bacterial sepsis in newborns aged 3–30 days admitted to the NICU (20 neonates with sepsis, 26 neonates without sepsis), and found a sensitivity of 98.6% and specificity of 88.9% for an optimum diagnostic cutoff value of ≥ 8.05 ng/mL, without statistically significant differences in comparison with CRP and serum amyloid. Kawczynski et al. [22] evaluated PCT and CRP in 48 newborn infants who suffered from nosocomial sepsis, reporting sensitivity of 89.6% for PCT and 66.7% for RCP at the onset of sepsis, that improved to 100% and 89.6% respectively 24 hours later. Unfortunately, only septic newborns where included, so it was no possible to calculate specificity. More recently, Vazzalwar et al [23] assessed PCT for the diagnosis of late-onset sepsis in 67 very low birth weight infants. At a PCT cutoff value of 1.0 ng/mL sensitivity was 97% and specificity 80%, while with CRP sensitivity was 72% and specificity 93%.

In the present study, the diagnostic reliability of serum PCT was modest, with sensitivities and specificities slightly higher than 80% at the time of suspicion of nosocomial infection. It should be noted that in the studies of Enguix et al. [20] and Chiesa et al. [21], a control group formed by asymptomatic infants without evidence of infection or with a diagnosis on admission easily differentiable from nosocomial sepsis was included, which may overestimate the reliability of a diagnostic test [26]. We studied a homogeneous group of neonates with clinically suspected nosocomial sepsis in which pre-established criteria for the definitive diagnosis of sepsis were applied, an approach that closely resembles the clinical scenario where the diagnostic test is intended to be used. On the other hand, because of the requirement of strict microbiologic criteria for the final diagnosis of sepsis, it may be possible that some cases of true bloodstream infection could not have been confirmed due to false negative blood cultures.

The use of PCT for the diagnosis of sepsis of vertical transmission is influenced by the physiologic peak of this marker during the first 48 h of life [9,27], but this phenomenon is obviated because nosocomial sepsis develops on a later time. It has been reported that elevations of PCT in neonates infected with CoNS are lower than serum PCT increases in sepsis caused by other pathogens [21]. In our study, serum PCT concentrations were significantly lower in the group of 22 patients with infections caused by CoNS than in the remaining 39 patients with infections of other etiologies. This is a relevant finding given the high frequency of infections caused by CoNS in the NICU.

Diagnostic reliability of serum PCT was not compared with CRP or other infection markers, such as leukocyte count, given that these laboratory tests were not standardized and were performed according to techniques of each participating hospital, and they were used to define sepsis. Although this may be considered a limitation of the present findings, the study was to assess the diagnostic usefulness of PCT as a single marker of neonatal sepsis of nosocomial origin. In addition, a comparative study would have required a bigger sample size in order to detect differences between infection markers.

Studies about diagnostic tests should be carried out in a patient sample that includes an appropriate spectrum of patients to whom the diagnostic test will be applied in clinical practice, and compared with a sufficiently reliable "gold standard" [26]. Unfortunately, an ideal "gold standard" for the definition of neonatal sepsis is not available and, for this reason, different definitions according to clinical, microbiologic, or laboratory criteria are used. Recently, the international pediatric sepsis consensus conference [28] modified the adult systemic inflammatory response syndrome (SIRS) criteria for children, incorporating pediatric physiologic variables for the subcategories of newborn, neonate, infant, child, and adolescent. However, premature infants were not included in the newborns group. Therefore, from the NICUs perspective, reference values of heart rate, respiratory rate, leukocyte count, and systolic blood pressure included in the definition of SIRS should be conveniently updated, as well as evidences (clinical, microbiologic, laboratory, radiologic) required to consider infection as the cause of SIRS. On the other hand, little guideline or consensus exists in literature for the differentiation between neonatal sepsis of vertical transmission and nosocomial sepsis, usually defined as early-onset and late-onset sepsis. The chronologic criteria, however, is inadequate for cases of early-onset nosocomial sepsis or late-onset bloodstream infection of vertical transmission that have a different profile of causative organisms and distinct therapeutic approach. An international consensus definition of these relevant aspects of pediatric sepsis will facilitate the performance of clinical studies in neonates with sepsis and the application of the results obtained in the daily practice.

## Conclusion

In this prospective multicenter study of 61 neonates with a definitive diagnosis of nosocomial sepsis, serum PCT concentrations showed a moderate diagnostic reliability for the detection of neonatal sepsis of nosocomial origin from the time of suspicion of infection. PCT is not sufficiently reliable to be the sole marker of sepsis, but would be useful as part of a full sepsis evaluation. Comparative studies with other markers of infection are needed as well as an adequate consensus definition of nosocomial neonatal sepsis to determine the real value of PCT in daily practice.

## Abbreviations

CI: confidence interval.

CoNS: coagulase-negative *Staphylococcus*.

CRP: C-reactive protein.

EPIDAT: epidemiological analysis of tabulated data.

NICU: neonatal intensive care unit.

PCT: procalcitonin.

ROC: receiver-operating characteristics.

SIRS: systemic inflammatory response syndrome.

SPSS: statistical package for the social sciences.

VLBW: very low birth weight.

## Competing interests

The author(s) declare that their have no competing interests.

## Authors' contributions

JBLS had primary responsibility for protocol development, outcome assessment, preliminary data analysis and writing of the manuscript.

DPS participated in the development of the protocol, literature search, performed the final data analyses, and had primary responsibility for writing of the manuscript.

VRS had primary responsibility for protocol development, patient enrolment and preliminary data analysis, supervised the design and execution of the study, and revised the manuscript for scientific content.

BFC and GDCC participated in the development of the protocol, patient enrollment, and contributed to the writing of the manuscript.

XKV, ENL, MGR, MSL, ABC, MMS, AUA, EAI, ACL, EMV, and BJC participated in the development of the protocol and patient enrollment, and approved the final draft.

## Pre-publication history

The pre-publication history for this paper can be accessed here:


